# Health-related quality of life for pediatric emergency department febrile illnesses: an Evaluation of the Pediatric Quality of Life Inventory™ 4.0 generic core scales

**DOI:** 10.1186/1477-7525-7-5

**Published:** 2009-01-29

**Authors:** Rakesh D Mistry, Molly W Stevens, Marc H Gorelick

**Affiliations:** 1 Department of Pediatrics, University of Pennsylvania School of Medicine, Philadelphia, PA 19104, USA; 2 Section of Pediatric Emergency Medicine, Department of Pediatrics, Medical College of Wisconsin, Milwaukee, WI 53226, USA; 3 Children's Hospital of Philadelphia, Division of Emergency Medicine, 34th Street and Civic Center Blvd., Philadelphia, PA 19104

## Abstract

**Objective:**

We sought to assess the validity and short-term responsiveness of the Pediatric Quality of Life Inventory™ 4.0 Generic Core Scales (PedsQL™) for febrile illnesses evaluated in the pediatric emergency department (ED).

**Design:**

Prospective cohort study of children 2–18 years discharged after ED evaluation for fever (≥ 38°C). Self-administered, parent-report of health-related quality of life (HRQOL) was assessed using the PedsQL™ Acute Version, a validated HRQOL instrument. HRQOL was measured on ED presentation and at 7–10 day follow-up. At follow-up, duration of fever, child functional impairment, missed daycare/school, and disrupted family unit functioning, were assessed.

**Results:**

Of 160 subjects enrolled, 97 (61%) completed the study; mean follow-up was 8.7 days. Mean total HRQOL score on ED presentation was 76.4; mean follow-up score was 86.3. Compared to subjects that returned to baseline, statistically significant differences in HRQOL were noted for those with prolonged fever, child functional impairment, and relapse. Significant correlation was observed between HRQOL at follow-up and days of daycare/school missed (r = -0.35, p = .003) and days of family disruption (r = -0.43, p < .001). Mean change in HRQOL within subjects, from ED visit to follow-up, was +9.8 (95% CI: 5.6–14.6). Effect size was 0.53, indicating moderate responsiveness.

**Conclusion:**

The PedsQL™ appears to be a valid and responsive indicator of HRQOL for short-term febrile illnesses evaluated in the ED.

## Introduction

Health-related quality-of-life (HRQOL) is an important patient-centered outcome, and the best available method for assessment of perceived health. HRQOL has yet to be assessed for many pediatric emergency department (ED) illnesses[1], although the need for such formal outcome measurements is a research priority for emergency medicine [2-4]. The lack of HRQOL data is largely a result of the absence of validated HRQOL instruments available for the ED setting.

When validating a HRQOL measure for the ED, there are several important considerations. The first is feasibility; the instrument should be easily administered, both in the ED and at the time of follow-up (FU). Another consideration is validity: the strength of the association between the candidate instrument and other relevant outcomes (construct validity). The instrument should also demonstrate responsiveness: the ability to show clinically and statistically significant changes in response to real changes in health status. An ideal HRQOL measure should be concise, easily administered, and exhibit both validity and responsiveness to ED illnesses. The Pediatric Quality of Life Inventory™ 4.0 Generic Core Scales (PedsQL™), Acute Version is a validated, reliable pediatric HRQOL instrument that has been used in other clinical settings [5-8]. The PedsQL™ has 23 items, takes less than 5 minutes to complete, and can be administered both in-person and via telephone. These characteristics make the PedsQL™ amenable for administration in the ED, and potentially useful for assessment of ED illnesses.

In this study, we examined the PedsQL™ for a typical, diverse population evaluated in the ED setting. Specifically, our objective was to determine the feasibility, validity, and responsiveness of the PedsQL™ for acute febrile illnesses. We selected febrile illnesses since they represent a common condition evaluated in the pediatric ED, accounting for over 20% of all ED visits[9,10], and have been targeted for outcome assessment by pediatric emergency medicine specialists[4].

## Methods

### Study design and setting

This was a prospective cohort study of febrile children evaluated in a large, tertiary-care pediatric ED.

### Study subjects and enrollment

Eligible subjects included children 2–18 years of age who: 1) presented to the ED on a study day; 2) had documented fever in triage (≥ 38°C), or presented with a primary chief complaint of fever; 3) and were discharged after physician evaluation. Subjects with chronic diseases (e.g. malignancy, sickle cell disease, immunosuppression) or prolonged fever (such as fever of unknown origin[11]) were excluded. In addition, patients that left prior to attending physician evaluation, were not accompanied by a legal guardian, were non-English speaking, or did not have a telephone were also excluded. The hospital's institutional review board approved the study.

Patient enrollment occurred on randomly selected study days distributed through July 2003 to July 2004. Randomization was performed in 2-month blocks throughout the year, to account for seasonal variability of pediatric febrile illnesses. Subjects were enrolled by trained clinical research assistants (CRAs), who were present 16 hours a day, 7 days a week during the study period. Patients presenting to the ED when CRAs were present were approached for enrollment after it was determined the child was to be discharged to home. Informed consent was obtained from the child's legal guardian; assent was obtained for children > 14 years of age. Subjects were enrolled irrespective of nurse triage assignments and discharge diagnoses. Routine ED standard of care was followed for all subjects, including diagnostic testing and therapeutic measures. A log of all patients approached for enrollment was maintained by CRAs; data for subjects not enrolled in the study were collected for comparison with enrolled subjects.

### Pediatric Quality of Life Inventory™ 4.0 generic core scales

The 23-item PedsQL™ Acute Version is a reliable, validated pediatric HRQOL instrument [12-14], offered in both child-report and parent-proxy report formats, with age-appropriate versions. The child report is available for children between 5–18 years, divided into the 5–7 (young child), 8–12 (child), and 13–18 year (adolescent) age groups. The parent-proxy forms may be used for children 2–18 years of age; with a 2–4 year (toddler) version. For this study, only the parent-proxy version was used. The PedsQL™ measures HRQOL in four domains: 1) physical functioning, 2) emotional functioning, 3) social functioning, and 4) school functioning. Items are scored from 0 to 4, with a score of 0 indicating "never a problem", and 4 representing "always a problem". Individual item scores are then converted using "reverse scoring" such that higher numeric scores reflect higher HRQOL. Typically, PedsQL™ scores are reported as the total of all items in the scale, reflecting a summary measure of HRQOL. However, subscale scores within each domain may be calculated, and summary scores in physical health (physical domain) and psychosocial health (combination of emotional., social, and school domains) can be evaluated, as well[12,15]. The PedsQL™ Acute Version, used in this study, utilizes a 7 day time-frame, and was created by the developers to assess the effects on HRQOL from short-term illnesses. The PedsQL™ Acute Version was administered according to the terms of the user agreement between the authors and distributors (MAPI Research TRUST).

### Data collection

Following enrollment, primary caregivers completed an age-appropriate, parent-proxy version of the PedsQL™. Medical records were reviewed to obtain demographic data (e.g. age, sex, race/ethnicity), and data pertaining to the ED visit, including date and time of the ED visit, chief complaints, triage temperature, and discharge diagnoses. Approximately 7–10 days after the initial ED visit, a member of the research team contacted the same caregiver present at enrollment via telephone to obtain follow-up (FU). The same age-appropriate, parent-proxy version of the PedsQL™ completed during the initial ED visit was then re-administered.

### Constructs for validity assessment

At the time of follow-up, caregivers were asked to report specific outcomes in several areas pertinent to the child and the family, to be used as constructs for PedsQL™ validity assessment. Constructs were selected from previously published ED studies of short-term outcomes and HRQOL, including prior assessments of fever [16], and acute asthma[17,18]. Child outcomes included duration of fever persistence, and duration of child functional impairments (i.e. activity, oral intake, sleep, behavior), and return to healthcare (Table 1). Duration of outcomes assessed was measured as days "abnormal"; the status of abnormal was evaluated via parental/caregiver perception of child and family morbidity. For cases which the caregiver reported abnormal at FU, the number of days from enrollment to FU was used for analysis; if the caregivers reported a range (i.e. "3 to 4 days") the lower number was used.

**Table 1:** Number of respondents, mean duration of child and family outcomes following the emergency department visit, and Spearman's Rho correlation between duration of outcomes and total PedsQL™ scores at follow-up (FU).

Outcome	N	Mean Duration of Outcomes (days, 95% CI)	Spearman's Correlation: Duration and PedsQL 4.0 at FU (95% CI)
Fever*^†^	92	4.53 (3.90–5.15)	*-.290 *(-.494 to -.085)
*Child Functional Impairment*			
Abnormal Activity*^†^	94	3.38 (2.85–3.91)	*-.412 *(-.588 to -.237)
Abnormal Oral Intake*^†^	95	3.27 (2.49–3.59)	*-.367 *(-.556 to -.178)
Abnormal Sleep*^†^	95	3.04 (2.49–3.59)	*-.400 *(-.584 to -.216)
Abnormal Behavior*^†^	95	3.41 (2.88–3.94)	*-.475 *(-.634 to -.315)
*Missed Daycare/School*	69	2.49 (1.85–3.13)	*-.348 *(-.560 to -.137)
*Caregiver Missed Work/School*	83	1.48 (0.94–2.02)	-.107 (-.323 to +.109)
*Family Disruption*	96	4.78 (3.77–5.80)	*-.427 *(-.592 to -.262)

*Caregivers were asked if the child had returned to normal for each outcome; if the child had returned to normal, the days they remained abnormal was then asked.

† Outcomes were adjusted to include subjects impaired at FU in the analysis, with the days to FU telephone call assumed as the final day of impairment.

Family outcomes reflected disruption of usual family unit functioning, including missed daycare or school for children, and lost school or work for primary caregivers. Days of disrupted family routine was collected as a global assessment of the effect of the child's illness on the family.

### Statistical analysis

Demographic characteristics, visit data, and outcome variables were summarized using standard descriptive statistics, and analyzed according to their parametric distributions. Total scale, summary, and domain scores for the PedsQL™ were calculated using the reverse scoring algorithm described by the developers[15], such that higher scores indicated improved HRQOL.

For the purposes of analysis, each child outcome was dichotomized to form "Child outcome status groups". Children febrile ≥ 7 days (equivalent to minimum time to FU) constituted the "Prolonged Fever" status group, while "Any Functional Impairment" reflected subjects reporting ≥ 7 days in one or more domains of functional impairment (activity, oral intake, sleep, behavior). "Return to Healthcare" included subjects making any non-scheduled return to healthcare, defined as any non-scheduled visits to the primary care physician (PCP) office, urgent care or ED. Caregivers were asked if a PCP or ED visits were recommended during the initial ED visit; for these subjects, the initial visit was considered scheduled from the ED, and any subsequent visits were considered non-scheduled. The child outcome status groups "Prolonged Fever", "Any Functional Impairment", and "Return to Healthcare" were further characterized as "poor outcomes". The proportion of subjects afflicted with each poor outcome was calculated, as were the proportions experiencing 0, 1, 2, or 3 poor outcomes.

We assessed construct validity under the hypothesis that children who remained abnormal at FU would have lower PedsQL™ 0 scores, compared to those who had returned to baseline. Therefore, mean PedsQL™ total scale scores at FU were compared between the each of the defined child outcome status groups. PedsQL™ scores were described using means and standard deviations; statistical comparisons were made using the independent samples *t*-test. In addition, PedsQL™ scores at FU were compared among groups of subjects experiencing 0, 1, 2, or 3 poor outcomes, using one-way ANOVA, and depicted graphically via box plots of means, interquartile ranges, and 95% confidence intervals. Construct validity was also assessed using Spearman's rho correlation between total PedsQL™ scores at FU and duration of child and family outcomes, under the hypothesis that patients with longer duration of fever, child functional impairment, and family unit functioning would have lower PedsQL™ scores at FU.

Responsiveness was analyzed using two methods. The mean change in total HRQOL score over our time frame of interest, from the initial ED visit to FU, was calculated for the outcome groups described above, as well as for the overall study population. Our hypothesis was children with prolonged fever, functional impairment, and return to healthcare, would have lower overall (or negative) changes in PedsQL™ total scores, when compared with those who had improved at FU. Mean change in HRQOL score was also compared among groups of subjects experiencing 0, 1, 2, or 3 poor outcomes, using ANOVA and box plots. As a second measure of responsiveness, we calculated the effect size, which assesses the ability of an instrument to detect changes in health status, by comparing the effect after treatment with the inherent variability of the score[19]. The overall mean change in PedsQL™ total score within the study population, from initial ED visit to FU, was analyzed by means of a paired *t*-test. The effect size was then calculated by dividing the overall mean change in score by the standard deviation of the score at baseline. As a point of reference, an effect size of 0.5 indicates moderate responsiveness of a given HRQOL instrument.

Sample size for the study was calculated based on the ability to detect an effect size of 0.5 or greater with a power of 90% and a α-level of 0.05. An estimated sample of 85 patients was needed. To account for an anticipated 20% loss to follow-up, our target enrollment was 106 patients.

The Statistical Package for the Social Sciences program, Version 12 for Windows was used for most statistical analyses. 95% confidence intervals (CI) for Spearman's rho correlation were created via bootstrap method[20], using *STATA Statistical Software: Release 7.0*[21].

## Results

### Feasibility and study population

During the study period, 160 of 197 (81.2%) subjects completed the initial PedsQL™ form and were enrolled into the study. Enrollment was in excess of the estimated sample size due to greater than anticipated loss to follow-up; consequently, an additional one-month block of study days was randomly selected early in the enrollment period. Of the 160 subjects enrolled, 97 (61%) were successfully reached for FU. For the 160 enrolled subjects, missing item response was 2.7% (95% CI: 2.2–3.1%); for the 97 subjects completing the study the missing item response was 2.9% (95% CI: 2.3–3.5%). Missing items were more likely to be present in school function domain compared with the rest of the instrument, at both enrollment (1.4% vs. 9.7%, *p *< 0.001) and follow-up (1.5% vs. 14.2%, *p *< 0.001).

The characteristics of patients completing the study are shown in Table 2. Mean follow-up occurred 8.7 days (range: 7–13) after the initial ED visit. Of note, there were no statistically significant differences in demographic and study characteristics between enrolled subjects and those not enrolled, or between patients completing the study and those lost to follow-up. Initial PedsQL 4.0 scores did not significantly differ between subjects enrolled and lost to follow-up.

**Table 2:** Characteristics of subjects completing the study (n = 97)

**Characteristic**	**Proportion** *(except where noted)*
Age (mean)	58.7 ± 40.1 months
Male	56%
Race/Ethnicity	
*White*	47.4%
*Black*	33.0%
*Hispanic*	18.6%
*Other*	1.0%
Triage Temperature (mean)	38.5 ± 1.1°C
Discharge Diagnosis*	
*Respiratory*	32%
*HEENT *^†^	30%
*Undifferentiated Febrile Illness*	19%
Time to follow-up (mean)	8.7 ± 1.8 days

*Discharge diagnoses not mutually exclusive, percentages represent top three diagnoses given and do not add to 100%.

^†^Head, Eyes, Ear, Nose, and Throat.

### Child and family outcomes

Caregivers reported a mean 4.53 days of fever for subjects, and at least 3 days of impairments in each area of functional impairment following the ED visit (Table 1). Subjects missed at least 2 days of daycare or school as a result of their illness, and caregivers missed at least 1 day of school or work, on average. After dichotomization into child outcome status groups, 44.4% of subjects reported at least one poor outcome (fever ≥ 7 days, functional impairment ≥ 7 days, return to healthcare), with 27.3% reporting one poor outcome, 11.4% reporting two poor outcomes, and 5.7% experiencing all three.

### PedsQL™ scores for study population

Mean PedsQL™ total scores for the study population at the initial ED visit and at FU are presented in Table 3. At enrollment, the mean PedsQL™ total scores was 76.5 ± 18.5, with a mean score at FU was 86.3 ± 5.2, yielding mean difference of 9.8 (95% CI: 5.6 to 14.6). Enrollment, follow-up, and change in physical and psychosocial summary scores were similar to total scores; among domain scores, social functioning was higher at both measurement points, while school functioning remained below total scores at both time points (Table 3).

**Table 3:** PedsQL™ total, summary, and subscale scores.

	Items	Mean PedsQL™ Scores in ED (± SD)	Mean PedsQL™ Scores at FU (± SD)	Δ PedsQL™ Scores ED to FU (95% CI)	Effect Size
*Total Score*	23	76.5 ± 18.5	86.3 ± 15.2	9.8 (5.6 – 14.0)	0.53
*Physical Summary*	8	74.4 ± 24.2	84.0 ± 21.6	9.6 (3.7 – 15.5)	0.40
*Psychosocial Summary*	15	78.4 ± 17.6	87.8 ± 14.7	9.3 (5.4 – 13.3)	0.53
Emotional	5	75.2 ± 23.2	86.5 ± 16.0	11.2 (5.7 – 16.7)	0.48
Social	5	85.0 ± 19.9	94.2 ± 15.4	9.2 (4.8 – 13.6)	0.46
School	5	69.9 ± 24.0	70.9 ± 28.8	3.9 (-5.6 – 13.1)	0.16

Abbreviations: ED, emergency department; FU, follow-up; SD, standard deviation

### Validity assessment

Mean PedsQL™ total scores at FU were significantly different within outcome status groups (Table 4). Children that remained febrile or with functional impairment, or had a non-scheduled return to healthcare, had significantly lower scores compared to their counterparts. Analysis of the individual summary and domain scores of the PedsQL™ revealed that physical functioning and school functioning scores were preferentially affected among subjects with prolonged fever or functional impairment, or with non-scheduled return to healthcare. Subjects with non-scheduled return to healthcare particularly exhibited significantly lower scores in the physical functioning domain (Table 4).

**Table 4:** Comparison of PedsQL™ total, summary, and subscale scores at follow-up, thin child outcome status groups.

	**Fever**			**Any Functional Impairment**			**Return to Healthcare**		
	
	≥ **7 days**	**< 7 days**	***P*-value**	≥ **7 days**	**< 7 days**	***P*-value**	**Yes**	**No**	***P*-value**
*Proportion with Outcome*	12.0%	88.0%	--	22.3%	77.7%	--	18.5%	81.5%	--
*PedsQL™ Scores at FU (mean ± SD)*									
Total Score	76.1 ± 22.4	88.2 ± 3.1	.011	76.4 ± 18.2	89.2 ± 2.9	< .001	73.8 ± 9.4	89.5 ± 1.9	< .001
Physical Summary	71.1 ± 22.3	85.7 ± 19.6	.166	70.9 ± 28.4	87.8 ± 17.6	.014	68.5 ± 29.8	87.1 ± 18.0	.024
Psychosocial Summary	80.6 ± 20.5	89.7 ± 11.6	.180	80.1 ± 16.3	90.1 ± 13.5	.005	77.4 ± 17.6	91.1 ± 10.6	.006
Emotional	81.8 ± 18.8	87.4 ± 15.3	.273	75.4 ± 18.3	89.8 ± 13.8	< .001	75.2 ± 20.9	89.6 ± 13.3	.014
Social	83.5 ± 24.6	96.7 ± 8.4	.106	89.2 ± 19.0	95.7 ± 13.9	.148	88.2 ± 20.8	97.0 ± 8.1	.106
School	65.7 ± 38.4	75.2 ± 24.1	.432	64.6 ± 33.1	72.8 ± 27.6	.375	56.3 ± 36.9	76.9 ± 23.0	.088

Abbreviations: FU, follow-up; SD, standard deviation

**Figure 1:**
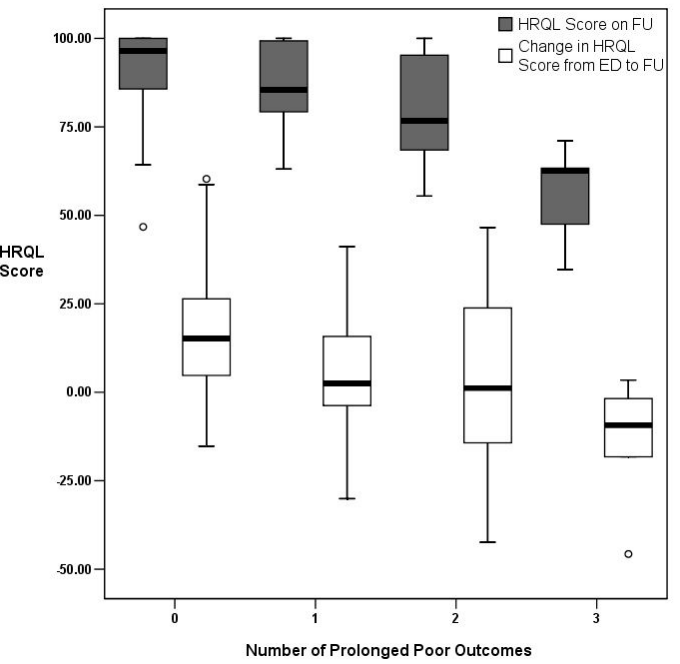
**Dose-response effect between the number of poor outcomes experienced by subjects and PedsQL™ Scores at follow-up (FU), and from the initial ED visit to FU**.

Mean PedsQL™ scores at FU significantly decreased with increasing number of poor outcomes reported by study subjects (Figure 1). For subjects reporting 0, 1, 2, and 3 poor outcomes, mean HRQOL scores at FU were 91.4 ± 11.2, 86.7 ± 12.4, 79.8 ± 16.4, and 55.8 ± 14.6, respectively (F = 13.88, *p *< 0.001).

There was moderate correlation between child and family outcomes and total HRQOL scores at FU. Individual measures of fever persistence, child functional impairment, and family unit functioning, significantly correlated with the PedsQL™ total scores at FU, with the exception of caregiver missed work or school (Table 1). The negative direction of the correlation reflects that the longer the duration of fever or abnormal functioning, the lower the PedsQL™ score at follow-up.

### Responsiveness

Mean change in PedsQL™ total scores between outcomes status groups was significantly different. Children that remained febrile or functionally impaired at FU, or made a non-scheduled return visit, had significantly lower changes in PedsQL™ scores. Patients who remained febrile or had a non-scheduled visit had a mean *negative *change in PedsQL™ total score (Table 5). Change in domain scores of the PedsQL™ again demonstrated that physical and school functioning scores were preferentially affected among subjects with prolonged fever, prolonged functional impairment, and with non-scheduled return to healthcare; subjects with prolonged fever and non-scheduled return to healthcare particularly demonstrated large negative changes in these domains (Table 5).

**Table 5:** Comparison ofthe change in PedsQL™ total, summary, and subscale scores from the emergency department visit to follow-up, within child outcome status groups

	**Fever**			**Any Functional Impairment**			**Return to Healthcare**		
	≥ **7 days**	**< 7 days**	***P*-value**	≥ **7 days**	**< 7 days**	***P*-value**	**Yes**	**No**	***P*-value**
*Proportion with Outcome*	12.0%	88.0%	--	22.3%	77.7%	--	18.5%	81.5%	--
*Change in PedsQL™ Scores ED to FU (mean ± SD)*									
Total Score	-5.1 ± 16.5	12.8 ± 20.5	.006	0.5 ± 24.0	12.6 ± 19.1	.016	-6.2 ± 24.4	13.4 ± 18.7	< .001
Physical Summary	-6.5 ± 27.8	12.1 ± 18.6	.045	-3.2 ± 35.9	13.4 ± 26.0	.019	-8.8 ± 40.3	12.5 ± 24.8	.050
Psychosocial Summary	-3.2 ± 13.9	12.4 ± 18.7	.009	2.9 ± 20.7	11.3 ± 18.7	.077	-4.4 ± 19.0	13.2 ± 17.5	< .001
Emotional	-0.45 ± 18.6	13.6 ± 27.8	.043	-3.0 ± 22.5	15.5 ± 26.9	.004	-9.7 ± 21.1	15.9 ± 26.3	< .001
Social	-3.3 ± 21.5	12.2 ± 20.1	.020	8.1 ± 29.3	9.5 ± 19.0	.787	5.3 ± 30.7	11.5 ± 17.7	.263
School	-19.3 ± 29.9	10.4 ± 29.7	.038	-3.6 ± 39.1	6.2 ± 32.1	.372	-17.5 ± 38.8	10.7 ± 25.9	.005

Abbreviations: ED, emergency department; FU, follow-up; SD, standard deviation

Our measure of total effect size was 0.53, indicating that the PedsQL™ demonstrates moderate responsiveness to change in health status for our study population. Moderate responsiveness was demonstrated among physical and psychosocial functioning; however, the effect size was poor for school function (Table 3.)

Mean change in PedsQL™ scores from ED to FU significantly decreased with increasing number of poor outcomes reported by study subjects (Figure 1). For subjects reporting 0, 1, 2, and 3 poor outcomes, mean change in HRQOL scores was +17.2 ± 18.2, +5.2 ± 19.5, +2.8 ± 25.9, and -14.3 ± 19.4, respectively (F = 5.70, *p *= 0.001).

## Discussion

HRQOL is an important patient-centered outcome, in that it provides an objective indicator of the patients' functional status and overall sense of well-being. HRQOL has been postulated as a method to populations are "at-risk" for poor outcomes[22], such as recidivism, future healthcare utilization, and higher healthcare costs[23,24]. The time required for completion and need follow-up assessment, represent important barriers for administration of HRQOL instruments in the ED setting, which is typified by brief, episodic encounters. Moreover, ED illnesses are short-term and diverse, while many HRQOL assessments are disease specific. These challenges have resulted in a lack of validated instruments for the ED, and a dearth of ED-based HRQOL investigations[22]. We believe our investigation is the first to assess a validated HRQOL instrument for a characteristic, heterogeneous pediatric ED illness, in the ED setting. Our results illustrate the PedsQL™ can overcome many of the barriers to ED assessment of HRQOL.

The results of our study demonstrate that the PedsQL™ is a practical and feasible for evaluation of short-term pediatric ED febrile illnesses. The brevity and ease of administration of the PedsQL™ allowed for enrollment and follow-up rates that resemble those of typical prospective studies conducted in the ED. Furthoermore, the missing item response rate in our study was quite, and was representative of other feasibility assessments of the PedsQL™[14].

The PedsQL™ demonstrated excellent construct validity for ED febrile illnesses: HRQOL scores were significantly lower at FU for children who remained febrile, functionally impaired, or relapsed to healthcare, compared with those who were asymptomatic or had not relapsed. Analysis of the subscales of the PedsQL™ demonstrated that impaired physical function of the child was particularly related to poor outcomes. This is sensible: physical impairments can certainly result from a febrile condition, and are visible to parents, leading them to return for further healthcare evaluation. Not surprisingly, school functioning domain was substantially affected, a valid finding: ill-children would be expected to have difficulty maintaining the level of concentration required to perform well in this setting. In addition to total and subscale analyses, increased days of fever, child functional impairment, and family unit functioning, were also significantly correlated with lower HRQOL scores. Moreover, HRQOL scores at FU decreased significantly with increasing numbers of reported poor outcomes, demonstrating a cumulative, dose-response effect. These encouraging findings support the construct validity of the PedsQL™ for short-term febrile illnesses in the ED setting.

The PedsQL™ also proved to be responsive to changes over a relatively brief time frame. Significantly smaller changes in HRQOL scores, from initial ED evaluation to FU, were exhibited for children that remained febrile, functionally impaired, or relapsed to healthcare. This statistically significant responsiveness was also present within the majority of measured domains of the PedsQL™. Responsiveness was greatest in the analysis or relapse to healthcare, again, consistent with the objectives of a HRQOL instrument; children with even worse perceived health would logically seek additional physician visits. Similar to our validity assessment, a dose-response relationship was also demonstrated between the change in PedsQL™ scores and increasing numbers of poor outcomes, consistent with statistical responsiveness to change in HRQOL. The responsiveness of the PedsQL™ in our study was also corroborated by the statistical measure of effect size, which persisted in the total and most of the subscale analyses.

Our evaluation generated results similar to those of prior studies of the PedsQL™, enhancing the validity of our findings. Population studies of the PedsQL™ have demonstrated that mean total scores for chronically ill and healthy populations are 73.1 ± 16.5 and 82.3 ± 15.6, respectively[14]. The mean total PedsQL™ score for our study population at enrollment, which occurred during the acute febrile illness, was similar to the mean for ill children with conditions frequently evaluated in the ED, including mild persistent asthma [25] and migraine headaches[26]. Similarly, the mean total score at FU, after resolution of the illness, was consistent with the population means for healthy children. Additionally, the mean change in PedsQL™ total score in our study population was nearly twice the calculated minimally clinically important change of 4.5 points[14].

Few previous studies have evaluated HRQOL for short-term ED illnesses, such as fever. In 2004, Gorelick et al. evaluated HRQOL following acute asthma exacerbations treated in the ED, using the Integrated Therapeutics Groups Child Asthma Short Form (ITG-CASF), a 10-item, asthma-specific HRQOL instrument[17]. The ITG-CASF was initially validated for use in chronic asthma; nevertheless, the authors found this instrument to be valid and responsive for acute asthma, using constructs similar to those in our study. This study was limited in that a disease-specific HRQOL instrument was used. In contrast, the PedsQL™ is a generic instrument, with the ability to assess HRQOL across a wide spectrum of conditions. This flexibility particularly suits the ED, where a variety of acute, short-term illnesses are evaluated. Shoham et al. evaluated HRQOL for another acute condition, community-acquired pneumonia[27], using an recurrent ENT infections HRQOL instrument. Using constructs similar to our study, and a short-time frame (21 days), significantly lower HRQOL scores were found for patients with community-acquired pneumonia, compared with controls. However, only 34.2% of subjects were enrolled on presentation to the ED, and the authors did not perform statistical analysis for validation or responsiveness of the HRQOL instrument. We were able to demonstrate responsiveness of the PedsQL™ over a shorter time frame, thereby strengthening the association between our constructs and HRQOL. Moreover, we were able to corroborate the validity and responsiveness of this tool using statistical methods.

In summary, the PedsQL™ exhibits feasibility, and statistically significant validity and responsiveness for a common, diverse ED illness. Our findings support potential utility of the PedsQL™ as an effective HRQOL measure for the pediatric ED setting. We feel our study of serves as an important starting point in assessment of HRQOL in the ED setting, and for short-term illnesses such as fever. As our ability to evaluate HRQOL in the ED becomes more advanced, investigators and clinicians will be able to use HRQOL and other patient-centered outcomes to assess their management decisions, including therapeutic interventions and discharge dispositions, to better benefit children and their families.

### Limitations

Our study does have several limitations. Despite multiple telephone attempts, 63 (39%) subjects were lost to follow-up, potentially introducing selection bias. However, demographic and study characteristics of subjects lost to follow-up were similar to those completing the study, and initial PedsQL™ scores did not differ, suggesting that lost subjects did not suffer from greater morbidity. The lack of follow-up also resulted in small numbers of subjects with "poor outcomes", our primary outcome measures, limiting the magnitude of our results. In our study, missing item responses were more likely to occur in the school functioning scale of the PedsQL™, introducing difficulty in statistical assessment of validity and responsiveness for this domain. This likely resulted because not all children required completion of all 5 scale items, since not all children were enrolled in daycare or school. Future ED studies of the PedsQL™ will need to focus on missing time responses to allow for a more complete assessment of the instrument. constructs and follow-up HRQOL scores were assessed by parental self-report, subjecting our results to observer and recall bias. To eliminate recall bias, in-person follow-up by a trained health professional would be necessary, which is neither feasible nor practical; furthermore, caregiver assessment of the child's health often is the impetus for caregiver behaviors; therefore, our results may actually represent a more clinically realistic situation. PedsQL™ assessments were only collected via the parent-proxy version. Ideally, validation should be accomplished using both the parent-proxy and child versions. We attempted to administer the child version in this study; however, < 50% of subjects were ≥ 5 years of age (the minimum age for the child report); this sample was too small to permit statistical analyses. Although there are Spanish versions of the PedsQL™, we only evaluated English-speaking patients, due to lack of translator availability.

## Conclusion

The PedsQL™ exhibited feasibility, construct validity, and responsiveness for short-term ED febrile illnesses, a commonly evaluated condition in the pediatric ED. The PedsQL™ is a generic HRQOL instrument, and is flexible enough to measure HRQOL across a diverse spectrum of diseases. Similar to febrile illnesses, there are numerous other acute, short-term conditions evaluated in the ED for which the PedsQL™ could be used. The flexibility, brevity, and sensitivity to short-term changes in HRQOL are desirable properties of the PedsQL™, making it a promising and potentially useful HRQOL measure for pediatric emergency medicine.

## Abbreviations

CI: Confidence interval; CRA: Clinical research assistant; ED: Emergency Department; FU: Follow-up; PCP: Primary care provider; QOL: Quality-of-Life; HRQOL: Health-related quality-of-Life.

## Competing interests

The authors declare that they have no competing interests.

## Authors' contributions

RDM, MWS, and MHG all participated in the conception and design and of the study. RDM and MWS were principally involved in the implementation and data collection phases of the study. RDM carried out all statistical analyses, under the guidance of MHG. RDM composed the manuscript; guidance and critical review was provided by MWS and MHG. All authors read and approved the final manuscript.
